# Reply to La Marca et al. Comment on “Naranjo-Bonilla et al. Retinal and Choroidal Effects of Continuous Positive Airway Pressure as Treatment for Sleep Apnea: Results at 12 Months. *Int. J. Environ. Res. Public Health* 2022, *19*, 12637”

**DOI:** 10.3390/ijerph20021141

**Published:** 2023-01-09

**Authors:** Pedro Naranjo-Bonilla, Rafael Giménez-Gómez, María Carmen Muñoz-Villanueva, Bernabé Jurado-Gámez

**Affiliations:** 1 Ophthalmology Department, Maimonides Biomedical Research Institute of Cordoba (IMIBIC), 14004 Córdoba, Spain; 2 Ophthalmology Department, University Hospital Juan Ramón Jiménez, 21005 Huelva, Spain; 3 Ophthalmology Department, Maimonides Biomedical Research Institute of Córdoba (IMIBIC), University Hospital Reina Sofía, 14004 Córdoba, Spain; 4 Primary Care Health Centre, Córdoba-Guadalquivir Health District, 14011 Córdoba, Spain; 5 Respiratory Department, Maimonides Biomedical Research Institute of Cordoba (IMIBIC), University Hospital Reina Sofía, University of Córdoba, 14004 Córdoba, Spain

Thank you for your interest [1] in our work [2].

In all choroidal thickness (ChT) images, the drawn lines are at the level of the RPE. In a recent study, we analyzed our intraobserver reproducibility for measurements of the choroid, and the intraclass correlation coefficients for measurements at the foveal, temporal, and nasal levels were 0.94, 0.87, and 0.80, respectively [3].

All follow-up images were captured using the follow-up setting.

Both the axial length and refractive defect were recorded. These data were not published because the scope of the journal was not strictly ophthalmological. The mean of axial length was 23.50 ± 0.91 mm. In any case, we were not comparing different subjects for which it would be useful to check for axial length differences between them. What we compared were data obtained in different follow-up visits, so we can assume that a given subject will have a similar axial length in each visit and that this measurement will have no effect on the results. 

Our group recently conducted a study in which we compared patients with severe obstructive sleep apnea (OSA) who did or did not undergo continuous positive airway pressure (CPAP) treatment to determine which effects are secondary to OSA and which are the consequence of CPAP [3]. 

In this study we observe patients in the group OSA + CPAP have a higher index apnea hypopnea (IAH) pre-CPAP than patients OSA no-CPAP (61.8 vs. 37.2 events/hour *p* < 0.001). We observe patients in the group OSA + CPAP have a higher foveal and temporal ChT pre-CPAP than OSA no-CPAP (271.29 vs. 232.16 µm, *p* = 0.018; 263.00 vs. 227.37 µm, *p* = 0.010). The more severe the IAH, the higher the ChT. After 3 months of follow-up, we do not observe changes in the OSA no-CPAP group. After 3 months of follow-up, we observed a reduction in temporal ChT in OSA + CPAP group (263.00 vs. 248.52 µm, *p* = 0.003). In conclusion, CPAP therapy could normalize ChT in severe OSA patients.

We refer to this article as the present study, as it is a continuation of the one designed to examine the effects of 12 months of CPAP therapy.

## Data Availability

The data presented in this study are available on request from the corresponding author.
